# Fragments of a Wheat Hevein-Like Antimicrobial Peptide Augment the Inhibitory Effect of a Triazole Fungicide on Spore Germination of *Fusarium oxysporum* and *Alternaria solani*

**DOI:** 10.3390/antibiotics9120870

**Published:** 2020-12-04

**Authors:** Larisa Shcherbakova, Tatyana Odintsova, Tatyana Pasechnik, Lenara Arslanova, Tatyana Smetanina, Maxim Kartashov, Marina Slezina, Vitaly Dzhavakhiya

**Affiliations:** 1 All-Russian Research Institute of Phytopathology, Bolshie Vyazemy, 143050 Moscow reg., Russia; beefarmer@yandex.ru (T.P.); linr-13@yandex.ru (L.A.); smetaneinatatyana@yandex.ru (T.S.); maki505@mail.ru (M.K.); dzhavakhiya@yahoo.com (V.D.); 2 Vavilov Institute of General Genetics RAS, Gubkina Str. 3, 119333 Moscow, Russia; omey@list.ru

**Keywords:** wheat hevein-like antimicrobial peptides, chemosensitization to agricultural fungicides, enhanced fungicidal effect, tebuconazole, synergy, *Fusarium oxysporum*, *Alternaria solani*, inhibition of spore germination

## Abstract

There are increasing environmental risks associated with extensive use of fungicides for crop protection. Hence, the use of new approaches using natural plant defense mechanisms, including application of plant antimicrobial peptides (AMPs), is of great interest. Recently, we studied the structural–function relationships between antifungal activity and five hevein-like AMPs from the WAMP (wheat AMP) family of *Triticum kiharae* Dorof. et Migush. We first discovered that short peptides derived from the central, N-, and C-terminal regions of one of the WAMPs (WAMP-2) were able to augment the inhibitory effect of Folicur^®^ EC 250, a triazole fungicide, on spore germination of the wheat pathogenic fungi, including *Fusarium* spp. and *Alternaria alternata*. In this research, we explored the ability of chemically synthesized WAMP-2-derived peptides for enhancing the sensitivity of two other *Fusarium* and *Alternaria* species, *F. oxysporum* and *A. solani*, causing wilt and early blight of tomato, respectively, to Folicur^®^. The synthesized WAMP-2-derived peptides synergistically interacted with the fungicide and significantly increased its efficacy, inhibiting conidial germination at much lower Folicur^®^ concentrations than required for the same efficiency using the fungicide alone. The experiments on co-applications of some of WAMP-2-fragments and the fungicide on tomato leaves and seedlings, which confirmed the results obtained in vitro, are described.

## 1. Introduction

The tomato (*Solanum lycopersicum* L.) is one of the highest-value vegetable crops for the human diet worldwide [1]. However, this crop is highly susceptible to a number of fungal diseases [2,3,4]. Among them, Fusarium wilt, caused by *Fusarium oxysporum* f. sp. *lycopersici*, and early blight, evoked by *Alternaria solani*, are the two most destructive for both open-field and greenhouse-cultivated tomatoes [5,6,7]. As with many crop plants, fungicidal treatments are frequently used as a reliable strategy to assure high yield and improved shelf-life of tomatoes. However, frequent or over-use of agrochemicals has a number of well-known drawbacks. Among them, there are the negative side effects on non-target species, contamination of the environment, and the potential for the development of resistance to fungicides in the target species. Triazoles, especially formulated together with strobilurins, are usually first tested in vitro and then commonly applied to tomato cropping systems in Russia and other countries [8,9,10,11,12]. These compounds belong to antifungal agents having a medium to high risk for the development of fungicide resistance [13]. In this regard, different strategies, such as breeding for resistance [14,15,16,17,18], various agricultural technologies, or biological control of agents, are applied along with fungicides to manage tomato diseases [5,19,20,21]. New environmentally friendly approaches, e.g., [22,23], are continuously being examined as alternate means to relying solely on fungicides. One of the more promising approaches is sensitization of pathogenic fungi to commercial fungicides. With this approach, the efficacy of antifungal agents is augmented, reducing the amounts of fungicide required for effectiveness. The sensitization-based approach also reduces the potential for the development of resistance to fungicides. This approach, called chemosensitization, involves co-application of non-fungitoxic or marginally toxic compounds to increase the sensitivity of pathogenic fungi to antimycotic agents. Such co-applications can produce a synergistic interaction by impairing the stress response system of the fungus, making it more vulnerable to fungitoxic formulations [24]. Chemosensitization can provide a high antifungal effect while reducing fungicidal dosages to levels wherein it would be ineffective if applied alone [24,25]. The discovery of effective chemosensitizing agents could be very useful in augmenting fungicidal efficacy against the above-mentioned tomato pathogenic fungi. Moreover, the use of such agents would reduce xenobiotic impact in tomato-growing areas and provide a more successful means of preventing the emergence of resistant strains in natural populations of plant pathogenic fungi [24,26,27]. Currently, chemosensitization and its implementation have been more intensively studied in human medicine than in agriculture. Nevertheless, it was shown that a range of various natural compounds of the plant and microbial origin as well as their artificially synthesized analogues are able to enhance the effect of some triazoles [24,28,29,30,31]. The discovery of new biomolecules, which might be used to augment the efficacy of modern industrial fungicides, could enlarge the variety of natural sensitizers and contribute to integrated management of Fusarium wilt and early blight of tomato.

Many properties of antimicrobial peptides (AMPs), such as a wide range of antimicrobial activity [32] and mode of action [33,34], make them promising as putative sensitizing agents [29]. Plants produce AMPs as a result of an evolutionarily conserved mechanism of innate plant immunity against pathogenic microorganisms. The mode of action of hevein-like AMPs remains poorly studied, but one of the generally acknowledged modes of action is binding of chitin and related oligomers of the fungal cell wall [35,36,37]. Interestingly, destabilization of the structural cell wall and/or membrane integrity is one of the mechanisms underlying the sensitizing activity of some natural compounds augmenting the sensitivity of fungi to triazoles and medical antimycotics [24,38,39,40]. WAMP-2 is one of the homologues of hevein-like cysteine-rich AMPs from a unique WAMP sub-family, which were discovered earlier in the kernels of wheat *Triticum kiharae* Dorof. et Migush [41] and other *Poaceae* [42,43]. Recently, we reported that short peptides derived from WAMP-2 potentiated the fungicidal effect of tebuconazole, a triazole fungicide, against five crop pathogenic fungi, including *F. culmorum*, *F. avenaceum*, and *Alternaria alternata*, causative agents of various diseases of wheat, barley, oat, and other economically important cereals. We tested non- to low-toxic concentrations of four WAMP-2 fragments (WAMP-N, WAMP-G1, WAMPG2, WAMP-C) and showed they synergistically interacted with tebuconazole to drastically increase the sensitivity of target pathogens to Folicur^®^ EC 250 (25% tebuconazole). The synergism provided higher or complete inhibition of spore germination in these fungi at dosages of Folicur^®^ ineffective alone [44]. The research, reported here, explored the ability of the four above-mentioned WAMP-2 fragments to enhance the inhibitory effect of Folicur^®^ on the conidial germination in *F. oxysporum* f. sp. *lycopersici* and to examine the sensitizing potential of WAMP-G1 and WAMP-C towards *A. solani*. Another important goal of the work was to confirm if in vitro antifungal effects were obtained in two different model plant-pathogen systems. In addition, we studied the antifungal activity of the WAMP-2-derived fragments against *A. solani*, and compared it with the fragments’ activity against *F. oxysporum* and the antifungal potency of the native WAMP-2. Thus, this study is a continuation of investigating the novel recently discovered property of hevein-like AMPs to increase the sensitivity of plant pathogenic fungi to agricultural fungicides. The overall goal is to provide a high efficacy of crop protection from diseases while greatly reducing dosages of these antifungal agents.

## 2. Results

### 2.1. Enhancement of the Fungicidal Effect by Folucur^®^ EC 250 by Co-Application with WAMP-2 Fragments In Vitro

Before studying the ability of WAMP-2 fragments corresponding to different regions of WAMP-2 to enhance the effect of Folicur^®^EC 250, we estimated the sensitivity of *F. oxysporum* and *A. solani* to these fragments. The antifungal activity of the fragments was measured by the level of inhibition of germination of fungal conidia.

In the first series of these experiments, we tested the inhibitory effect of the fragments against *F. oxysporum* and compared the inhibitory activity of the fragments to each other and to the entire WAMP-2 peptide. Microconidia of the pathogen were treated to each of the WAMP-2 fragments taken at three or four concentrations ranging between 50 and 200 (WAMP-N, WAMP-G2), 100 and 400 (WAMP-G1), or 50 and 400 (WAMP-C) µg/mL. The prior research showed these concentrations were marginally or low toxic for the fungus [44]. After the treatments, the rate of germinated conidia was determined, expressed as the percent relative to the total number of conidia counted (500 ones per treatment). Effective doses (EDs) providing a 50% or 99.9% inhibition were calculated by probit analysis. The performed analysis confirmed our previous data on the antifungal activity of WAMP-2-derived fragments towards the Fusarium wilt agent. The obtained results showed *F. oxysporum* was significantly more sensitive to WAMP-N and WAMP-G2 than to other peptide fragments (Table 1). The results also indicated the entire WAMP-2, the activity of which was determined earlier under the same conditions [44], had a lower inhibitory effect than either of these more active fragments. WAMP-G1 possessed no antifungal activity towards this fungus; the minimum fungicidal concentration (MFC) exceeded 10,000 µg/mL. WAMP-C possessed almost the same inhibitory activity as the full wheat hevein-like WAMP-2 (Table 1).

In the next experiments, we assessed the effect of WAMP-G1 and WAMP-C (at 100, 200, and 400 µg/mL), the least active fragments against *F. oxysporum*, on conidial germination in *A. solani*. These pathogens were found to differ in their sensitivity to WAMP-G1 and WAMP-C, and *A. solani* was significantly more responsive to both WAMP-2-derived peptides. (Table 1).

Unlike its inactivity against *F. oxysporum*, WAMP-G1 impeded germination of *A. solani* macroconidia with 50% efficacy at a concentration equal to the ED_50_ of WAMP-2. At the same time, WAMP-C inhibitory activity against *A. solani* at the ED_50_ level significantly exceeded the ED_50_ of the native peptide for *F. oxysporum*, and MFC was two times lower than against *F. oxysporum*.

In general, the synthesized fragments, inhibiting the spore germination by 50% in the range of hundreds or tens of hundreds of micrograms per mL, demonstrated a very low or nominal toxicity towards the target pathogens in comparison to our ED_50_ calculations for Folicur^®^ against *A. solani* (0.2 ± 0.04 µg/mL) and *F. oxysporum* (0.2 ± 0.02 µg/mL). It should be noted that the vast majority of known natural and synthetic compounds, like the WAMP-2 fragments, possess an order to several orders of magnitude lower antifungal activity than commercial antimycotics [24].

**Figure 1:**
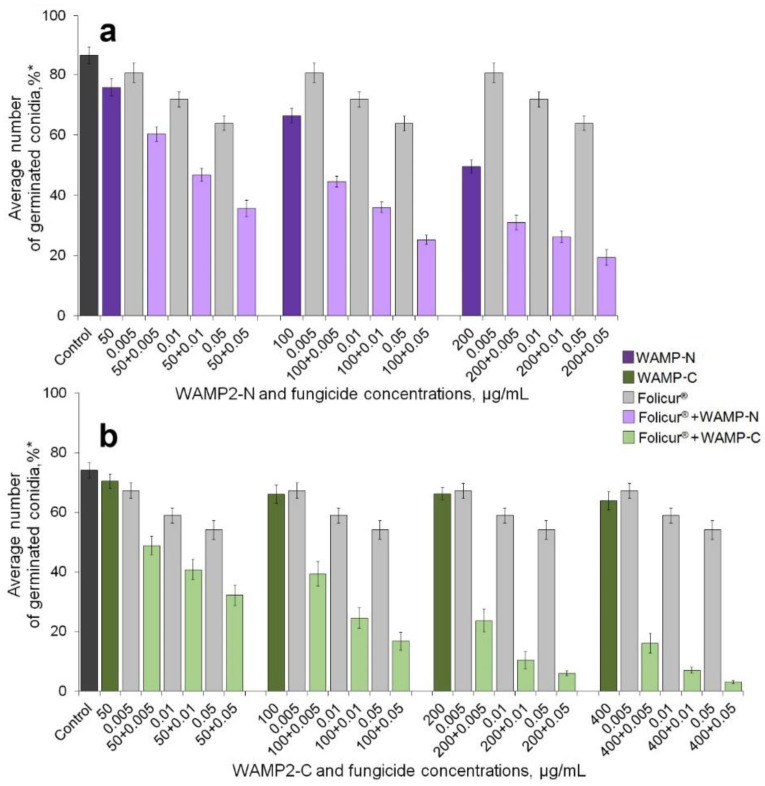
Inhibitory effect of Folicur^®^ EC 250 on the germination of *Fusarium oxysporum* microconidia exposed to (**a**) N-terminal (WAMP-N), (**b**) C-terminal (WAMP-C) fragments of a wheat hevein-like antimicrobial peptide WAMP-2, (**a**,**b**) Folicur^®^, alone, and this fungicide in combination with (**a**) WAMP-N or (**b**) WAMP-C. * The average number of germinated conidia is expressed in percent relative to the total number of conidia counted in controls and treatments. Suspensions of conidia germinated in sterile distilled water (SDW) were used as controls. Columns represent the means calculated for three experiments (100 conidia in one replication of a treatment, 5 replications per treatment in each of three experiments); in total, 1500 conidia were examined per treatment. Experiments were carried out with independently synthesized WAMP-N and WAMP-C peptides on conidia sampled from three plates with the pathogen colonies grown independently for each of the experiments. Y-bars indicate standard deviations (SD). Significance of differences of means between treatments and controls at *p* ≤ 0.05 was determined using a *t*-test for independent variables (STATISTICA v. 6.1, StatSoft Inc.). For each concentration combination, Ee values calculated by Limpel’s formula (see Material and Methods, 4.7), and observed Er values are presented in Table 2.

**Figure 2:**
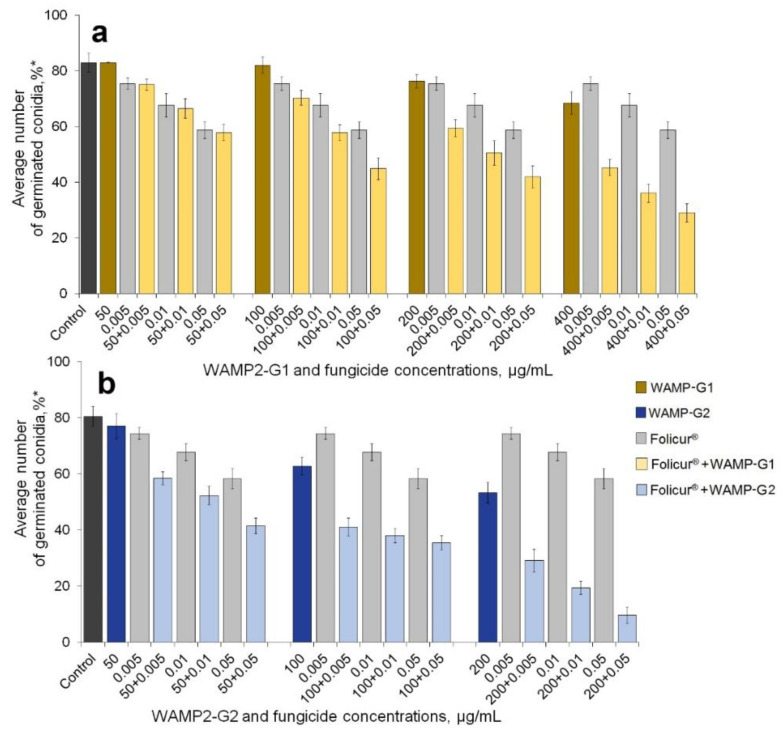
Inhibitory effect of Folicur^®^ EC 250 on germination of *Fusarium oxysporum* microconidia exposed to (**a**) WAMP-G1 or (**b**) WAMP-G2 derived from the central regions of a wheat hevein-like antimicrobial peptide WAMP-2, (**a**,**b**) to Folicur^®^, alone, and to this fungicide in combination with (**a**) WAMP-G1 or (**b**) WAMP-G2. * The average number of germinated conidia relative to the total number of counted conidia is expressed in percent. Microconidia germinated in SDW were served as controls. The means are calculated for 1000 conidia per treatment (*n* = 500 per treatment in each of three experiments) Y-bars indicate SD at *p* ≤ 0.05. For additional explanations, see Figure 1.

**Figure 3:**
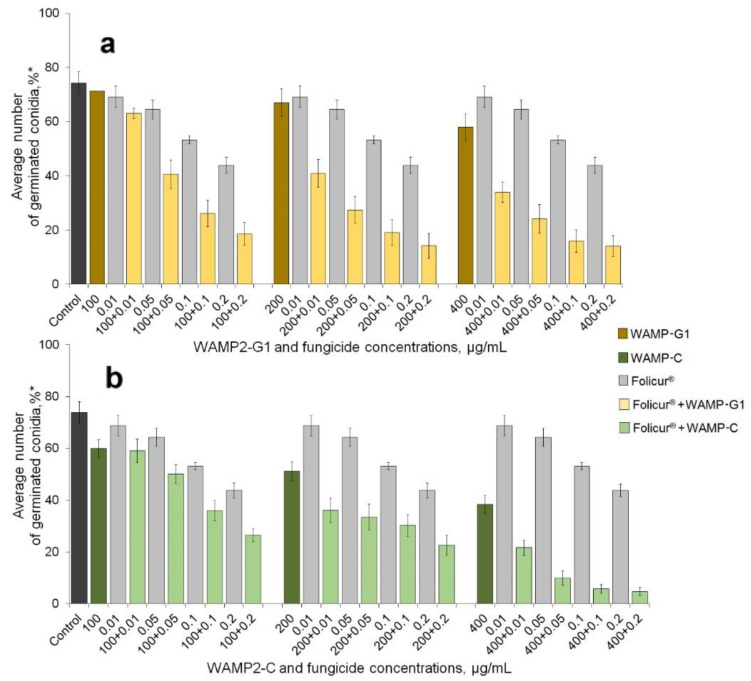
Enhancement of the inhibition of *A. solani* conidial germination after treatments with Folicur^®^, EC 250 combined with (**a**) WAMP-G1, or (**b**) WAMP-C compared to (**a**,**b**) Folicur^®^ alone. * Percentage of germinated conidia of the total number of conidia counted. Controls are *A. solani* conidia germinated in SDW. See also the explanations in the Figure 1 caption.

We next evaluated the ability of WAMP-2 fragments to augment inhibition of spore germination by Folicur^®^ against both fungi. We tested the activity of the fragments, when applied alone, at concentrations that produced marginal (1–9%), low (10–39%), or 40–50% inhibition of the spore germination (see Figure 1, Figure 2 and Figure 3).

In all cases, observed levels of inhibition in co-applications (Er) significantly exceeded the effects of the fungicide alone. Additionally, in all cases except one (WAMP-G1 100 µg/L and Folicur 0.005 µg/L), Er values exceeded Ee values (Table 2). These observations clearly showed the augmented fungitoxicity of co-applications was synergistic in nature.

Folicur^®^ was used at the sub-fungicidal dosages, the efficacy of which did not exceed 30% (*F. oxysporum*) or 45% (*A. solani*). The assays were performed in 96-well microtiter plates, using a “checkerboard” design. Fungal conidia were treated with either Folicur^®^ or peptide fragments, each alone, and, in parallel, were subjected to combined treatments wherein the fungicide and one of the WAMP-2-derived peptides were mixed in the same concentration as when used alone. In general, such co-applications resulted in a noticeable augmentation of the fungicidal effect against *F. oxysporum* (Figure 1 and Figure 2) and *A. solani* (Figure 3). However, enhancement potency varied depending on the pathogen, the WAMP fragment, and co-applied concentrations of the WAMP-2 fragments and Folicur^®^.

Co-application of the terminal fragments WAMP-N and WAMP-C with Folicur^®^ resulted in an evident decrease in the number of germinated *F. oxysporum* conidia (Figure 1). To confirm that the augmented antifungal effect was due to a synergistic interaction between the fungicide and WAMP-2 fragments, and to reveal combinations of fractional concentrations resulting in the synergy, inhibitory effects observed in co-applications (Er) were compared with calculated additive effects of the fungicide and each peptide fragment (Ee) (Table 2).

In all cases, the inhibition observed in treatments with Folicur^®^ combined with terminal fragments significantly exceeded not only the inhibitory effects of the fungicide alone but also the calculated additive effects (Table 2). These results suggested that the fungicidal effect was enhanced due to a synergy between components in the Folicur^®^/WAMP-N (Figure 1a) and Folicur^®^/WAMP-C (Figure 1b) combinations. No statistically significant difference in sensitizing activity between these peptides was reveled when they were applied at 50 µg/mL in combination with the fungicide at 0.01 µg/mL. Additionally, there were no differences in the sensitizing activity of WAMP-N and WAMP-C when they were applied at a range of 50 to 200 µg/mL together with the lowest Folicur^®^ dose (0.005 µg/mL). In the same range of dosages, the C-terminal fragment co-applied with higher Folicur^®^ concentrations (0.01 or 0.05 µg/mL) enhanced the fungicidal activity more effectively than WAMP-N (Figure 1 and Table 2). Notably, co-application of Folicur^®^ at 0.01 or 0.05 µg/mL and WAMP-C at 400 µg/mL resulted in almost complete suppression of the pathogen germination (Figure 1b and Table 2).

Among all synthesized fragments, WAMP-G1 derived from the central region of the WAMP-2 region demonstrated the highest degree of enhancing fungitoxicity of Folicur^®^ on spore germination of *F. oxysporum* (Figure 2 and Table 2). In most cases, the fungicide and WAMP-G1 interacted in a synergistic manner. Their combinations provided a higher inhibitory effect compared to Folicur^®^ alone (Figure 2a and Table 2). However, co-applications of WAMP-G1 at a fractional concentration of 50 µg/mL resulted in no synergism (Figure 2a). In addition, one of the concentration combinations with higher WAMP-G1 doses produced an additive effect (Table 2).

The sensitizing efficacy of WAMP-G2 towards *F. oxysporum* was comparable rather to the efficacy of the spatially more distant N-terminal fragment (WAMP-N) [35] than that of the neighbor WAMP-C (Table 2). In general, a common trend observed for all synthesized fragments in augmenting the fungicidal effect was a positive correlation with fractional concentrations (Figure 1, Figure 2 and Figure 3). However, this trend was less apparent with the peptides from the central WAMP-2 region (Figure 2).

Augmentation of the antifungal efficacy was also found if Folicur^®^ was co-applied with WAMP-G1 or WAMP-C against *A. solani* (Figure 3). In tests involving this pathogen, the synergy was most often observed when combining fungicide with WAMP-G1 (Figure 3a and Table 3).

Co-application of WAMP-C and the fungicide against *A. solani* resulted in an additive effect in 5 of 22 concentration combinations, while others were synergistic (Figure 3b). The response of *A. solani* to combined treatments, which included WAMP-C at concentrations of 100 or 200 µg/mL, did not depend on the Folicur^®^ fractional concentrations except for three mixes (100 + 0.1, 100 + 0.2, and 200 + 0.2 µg/mL). As in case of *F. oxysporum*, exposure of conidia to the mixes containing WAMP-C at a concentration of 400 µg/mL provided almost complete suppression of *A. solani* germination.

### 2.2. Study of the Sensitizing Activity of WAMP-2 Fragments Using a Wilting Test on Detached Seedlings and a Leaf Disk Assay

In order to confirm that the sensitization of *A. solani* and *F. oxysporum* to the fungicide by the synthesized peptide fragments might be subsequently realized on plants, we determined the effectiveness of the sensitization of these two pathogens to Folicur using live tomato tissue assays. These experiments were performed using detached tomato seedlings and leaf disks treated with combinations of Folicur^®^ mixed with WAMP-2-derived oligopeptides. For these experiments, WAMP-C and WAMP-G1, which showed different antifungal and sensitizing activities towards these two pathogens under in vitro conditions, were chosen. The C-terminal oligopeptide was the most active sensitizer of *F. oxysporum* compared to all other fragments, while WAMP-G1 combined with the fungicide provided mainly the synergistic effect towards *A. solani*.

**Figure 4:**
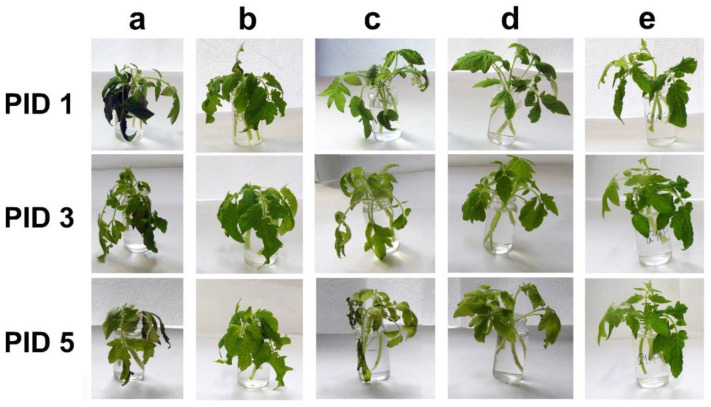
Representative photos showing results of the *F. oxysporum* wilting test on detached tomato seedlings. Seedlings were inoculated by the tomato wilt pathogen *F. oxysporum* f. sp. *lycopersici*, F37 by immersion of stems in a suspension of conidia, 3 × 10^6^ (control) or in portions of the same suspension supplemented with either Folicur^®^ only (at 0.05 or 0.005 µg/mL) or fungicide at 0.005 µg/mL combined with WAMP-2 derived peptides (each at 200 µg/mL). (**a**) Control treatment (only *F. oxysporum*); (**b**) Folicur^®^, 0.05 µg/mL; (**c**) Folicur^®^, 0.005 µg/mL; (**d**) Folicur^®^, 0.005 µg/mL + WAMP-C; (**e**) Folicur^®^, 0.005 µg/mL + WAMP-G1. PID 1, PID 3 and PID 5 are the first, third, and fifth post-inoculation days, respectively.

To inoculate seedlings with *F. oxysporum*, a conidial suspension was prepared and divided into three portions. One portion was diluted with an equal volume of SDW to serve as a pathogen-inoculated control. The second portion was mixed with an equal volume of aquatic Folicur^®^, and the third portion was added to the fungicide combined with either WAMP-C or WAMP-G1. Final concentrations in each portion included microconidia at 3 × 10^6^/mL, peptide fragments at 200 µg/mL, and Folicur^®^ at two concentrations (0.005 and 0.05 µg/mL). Stems of tomato seedlings with four or five true leaves were detached from roots and placed in the prepared suspensions for two days, so that only stems, but not leaves, remained submerged. Inoculated seedlings were incubated with the detached seedlings in Knop nutrient solution for the next three post-inoculation days (PIDs). To confirm that WAMP-derived peptides possessed no phytotoxicity, two other groups of seedlings were preliminary incubated in SDW or that added with the peptides. These preliminary assays showed WAMP-C and WAMP-G1 had no phytotoxicity at the used concentration (Figure S1). Detached seedlings incubated in the control suspension started to wilt one day after inoculation (Figure 4a). Wilting of seedlings exposed to Folicur^®^ started on PID 3, and all seedlings treated with the fungicide at the lower dosage of 0.005 µg/mL wilted at PID 3 (Figure 4c). The combined treatments delayed wilting up to the end of the experiment (Figure 4d,e) and provided an anti-wilt protection equivalent to that provided by a 10-fold higher dosage of Folicur^®^ (0.05 µg/L) throughout the observation period (Figure 4d,e). Except for a light yellowing, no other Fusarium wilt symptoms or any observable deterioration were noticed on these seedlings compared to those incubated in SDW or submerged in peptide solutions.

The ability of WAMP-G1 to enhance the fungicidal effect against *A. solani* was also shown using the tomato leaf disk assay performed according to Alan and Earle [32] with some modifications (see materials and methods). Conidia of *A. solani* were incubated in a suspension combining WAMP-G1 and a sub-fungicidal dose of Folicur^®^, or a suspension containing only Folicur or WAMP-G1 at the same respective concentration used in the combined suspension.

**Figure 5:**
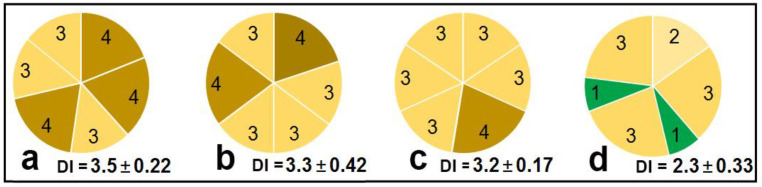
Diagrams illustrating results of the tomato leaf disk assay that included inoculations of disks with *A. solani* conidial suspensions in (**a**) water; (**b**) Folicur^®^ (0.05 µg/mL); (**c**) WAMP-G1 (200 µg/mL); and (**d**) mixture containing this fungicide and WAMP-G1 at the same concentrations as in (**b**,**c**). Numbers in the diagram sectors indicate scores for each of six leaf discs assayed. DI is average scoring of the damage (mean ± SE).

Drops of the test suspensions containing Folicur/peptide-treated or SDW-treated (control) conidia were applied to the adaxial side of leaf disks placed on the surface of water agar in Petri plates. After inoculation, the plates were transferred to a climate chamber for disease development. At the 9th day of incubation, the damage symptoms on leaf disks were scored. The lesions on control disks developed beyond inoculation drops. Almost the entire surface yellowed on three of the six treated disks. Similar symptoms were also observed on leaf disks exposed to Folicur^®^, alone, and WAMP-G1, alone. After such treatments, the calculated average disease severity index (DI) did not differ from that in the control (Figure 5a–c). In contrast to this, the combined treatment mitigated leaf damage. Lesions on disks inoculated with fungal conidia, which were pre-incubated in the WAMP-G1/fungicide mixture, were less expressed compared to both control and symptoms observed in the two previous variants. Some of the lesions were small and located only within the inoculation area, DI did not exceed 2.6 making 2.3 on average, i.e., was 1.4–1.5 times lower compared to the three other DI values (Figure 5d).

## 3. Discussion

Along with the structural and functional similarities with other hevein-like AMPs, WAMPs have a number of specific characteristics [45,46], display an in vitro inhibitory activity towards various microorganisms including plant pathogenic fungi [36,47,48], and are able to inhibit a fungal effector fungalysin that cleaves plant chitinases. The efficacy of the inhibition was shown to depend on the amino acid residue at position 34 in the WAMP polypeptide chain. Recently, we have shown that a wheat WAMP homologue, WAMP-2, containing lysine at this position, possessed relatively weak activity against *F. culmorum* but was able to suppress the spore germination in *F. oxysporum* and *A. alternata*. We also found that the terminal (WAMP-N, WAMP-C) fragments and the oligopeptides derived from the central region of the WAMP-2 molecule (WAMP-G1, WAMP-G2) significantly enhanced the sensitivity of some soil-borne and foliar cereal-damaging fungi to a triazole fungicide, tebuconazole, formulated as Folucur^®^EC 250 [44], while WAMP-2 was inactive as a sensitizer. 

To continue and develop this research, we investigated the ability to sensitize *F. oxysporum* and *A. solani* to Folicur with the aforementioned WAMP-2-derived oligopeptides in two model plant-pathogen systems. The obtained results confirmed our previous findings. All tested oligopeptides augmented the inhibitory efficiency of the fungicide towards conidial germination in these fungi. Results also showed, in general, the sensitizing activity of synthesized N- and C-terminal fragments was greater compared to the sensitizing activity of peptides from the central region of WAMP-2 (Figure 1 and Figure 3). Furthermore, our prior experiments with cereal-damaging fungi, using a similar in vitro assay as used here, also showed WAMP-N and WAMP-C had the highest synergy with tebuconazole [44], as obtained here with tomato pathogens (Figure 1 and Figure 3). Interestingly, WAMP-G2, quite effective as the sensitizer of *F. culmorum* and *F. avenaceum* [44], demonstrated the lower sensitizing activity towards *F. oxysporum* (Figure 2). As in the case of wheat pathogenic *F. culmorum* and *F. avenaceum*, WAMP-G1 less actively sensitized the wilt pathogen, *F. oxysporum*. At the same time, WAMP-G1 synergistically enhanced the Folicur^®^ effect against *A. solani*, although this oligopeptide was earlier found to provide no sensitization of *A. alternata* [44]. 

We also made the first step to validate whether the sensitization discovered in vitro allows augmentation of the antifungal effect on plants. With the plant assays, we tested the two more promising fragments, WAMP-G1 and WAMP-C, at peptide/fungicide concentrations of 200/0.005 and 200/0.05 µg/mL, having similar Ers values (from 48% to 60%) (Table 2 and Table 3). Folicur^®^, alone, was tested at 0.005 and 0.05 µg/mL (Figure 4 and Figure 5). The results obtained using the detached tomato seedlings inoculated with *F. oxysporum* microconidia, which were treated with Folicur^®^ combined with either WAMP-G1 or WAMP-C (Figure 4), confirmed the enhanced fungicidal effect observed in vitro. The augmented protective effect of Folicur^®^ mixed with WAMP-G1 was also confirmed towards *A. solani* by the tomato leaf disc assay (Figure 5). Since promising results of in vitro tests are not always reproduced in plant tissue assays, our results warrant further investigations for expanded assay conditions, such as greenhouse or field experiments.

In spite of quite limited studies related to chemosensitizaton of pathogenic fungi to agricultural fungicides using AMPs [25,29,44,48], the promise of some microbial and plant AMPs for augmentation of the antifungal effect is already acknowledged. The discovery of the synergism of WAMP-2-derived peptides with one of the triazole fungicides, and the enhanced antifungal effect against two harmful tomato pathogens coincide with the data of other authors. For instance, Kim et al. [29] reported sensitizing activity of bacterial AMPs, produced by non-ribosomal biosynthesis, co-applied with triazoles to control *F. graminearum* in vitro and under greenhouse or field conditions. In addition, interaction with fungicides resulting in synergistic reduction of biofilm formation in *Candida albicans* was shown for radish defensins [48]. There are also some reports that the protective activity of a short peptide fragment tested against plant pathogens can be equal to or greater than the activity of the intact source peptide, e.g., [49].

Triazoles, belonging to DMI fungicides, inhibit 14-α-demethylase from participating in the synthesis of ergosterol, the principal sterol in fungal cell membranes [50]. On the other hand, WAMPs degrade cell wall integrity. Thus, the synergy between WAMP-2-derived peptides and Folicur^®^, first discovered in our previous study and also revealed in this work, is evidently attributed to the fact that these sensitizing agents attack different targets in the tested plant pathogens. This resulted in a severe weakening of the pathogen vitality and a drastic enhancement of the fungicidal effect.

It should be mentioned that triazole fungicides control the development of various plant pathogenic fungi by effective suppression of their growth. It is commonly held that they are unable to inhibit germination of their spores with the same efficacy, since fungal spores already contain ergosterol. However, our results suggest that combining triazoles with WAMP-2 fragments may increase the efficiency of these fungicides against spore germination, the key stage of the plant infection process.

A further factor to consider, in addition to enhancing the fungicidal effect using WAMP-2 fragments, is the potential for reducing the effective dosage levels of triazoles required for fungal control. This could mitigate pesticide impact on the environment. Furthermore, resistant strains pathogenic to tomato will probably be better controlled with triazoles without increasing their dosages because of significant enhancement of the fungicidal effect by the addition of the sensitizing peptides.

However, so far, our results show only the potential for the synthesized WAMP-2 fragments to enhance the effectiveness of industrial fungicides in agricultural practice. These results should be confirmed not only in plant-pathogen model systems but also by experiments with plant treatments. As compared to recombinant WAMP-2, commercial production of which would be complicated, obtaining short peptides via direct chemical synthesis is practicable. Testing the sensitizing activity of other plant AMPs, and their fragments, is necessary to select the most active sensitizers and to understand the interrelationship between the peptide structure and sensitizing activity. In addition, such investigations could contribute to our knowledge of chemosensitization mechanisms. Another important issue is study of the range of action of the revealed sensitizers. Triazole fungicides giving the most effective combinations with WAMP-2 fragments, and crop pathogens most responsive to the combined treatments should be revealed using various plant-pathogen model systems, and also under greenhouse and field conditions.

## 4. Materials and Methods 

### 4.1. Tomato Pathogens

*Fusarium oxysporum* f. sp. *lycopersici* (Sacc.) W.C. Snyder and H.N. Hans (strain F37), causing wilt of tomato, was obtained from the curated collection of plant pathogens of the Laboratory of Physiological Plant Pathology at the All-Russian Research Institute of Phytopathology (ARRIP). *Alternaria solani* (Ell. & Mart.) L.R. Jones and Grout. (strain MO-VNIIF-9-2018), a causative agent of tomato early blight, isolated from naturally infected plants cultivated on the ARRIP experimental field in the Moscow region, was provided by the ARRIP Department of Potato and Vegetable Diseases. Stock cultures of the pathogenic strains were maintained on potato dextrose agar (PDA) slants in the dark at 25–26°C by sub-culturing after each four-six weeks.

To obtain spore-producing colonies and prepare spore suspensions, the pathogens were re-cultured on PDA in 9-cm Petri plates under the above conditions for 7 (*F. oxysporum*) or 14 (*A. solani*) days. Fungal conidia were collected from the colony surfaces and separated from mycelial debris, as described earlier [22]. Production of macroconidia of *A. solani* was additionally stimulated by flooding mycelia with sterilized ice water followed by short-time UV-irradiation [51], then drying for 3–5 days under a diffused light (16-h illumination period) at ambient temperature [32]. Concentrations of conidia in the suspensions were determined using a hemocytometer.

### 4.2. Production of WAMP-2-Derived-Peptides and WAMP-2

Four peptides corresponding to N- and C-terminals, as well as central regions of the peptide WAMP-2 (Table 4), were produced by a solid-phase chemical synthesis using Fmoc chemistry, and then purified by RP-HPLC. The identity of the synthesized peptides was confirmed by mass spectrometry. To compare the antifungal activity of the synthesized fragments with the activity of the whole peptide, recombinant WAMP-2 was produced in *E. coli* BL21 (DE3) and purified by HPLC on a Luna C8 column (4.6 × 150 mm, Phenomenex) as described earlier [47,52]. MALDI-time-of-flight mass spectrometry on an Ultraflex MALDI-TOF mass spectrometer (Bruker Daltonics, Bremen, Germany) in linear or reflector positive-ion mode using alpha-cyano-4-hydroxycinnamic acid as a matrix, and also N-terminal Edman sequencing were used to confirm peptide purity. The purity of peptides was >95%. The molecular weights of WAMP-2-derived oligopeptides were calculated using ExPASyProtParam tool [53].

Freeze-dried peptide preparations were stored at −20°C and dissolved in sterilized distilled water (SDW) on the day of testing their antifungal or sensitizing activities.

To compare the antifungal activity of synthetic fragments with that of WAMP-2, WAMP-2 recombinant peptide was obtained as fusion protein with thioredoxin in *E. coli* BL21 (DE3), as described earlier [47,51]. The fusion protein was isolated by affinity chromatography on TALON Superflow resin and cleaved with cyan bromide. The target peptide was purified by HPLC on a Luna C8 column (4.6 × 150 mm, Phenomenex). Peptide purity was confirmed by MALDI-time-of-flight mass spectrometry and N-terminal Edman sequencing.

### 4.3. Fungicide

Folicur^®^ EC 250, a commercial pesticide formulation (Bayer AG, Leverkusen, Germany), containing 25% tebuconazole as the active ingredient, was used for both in vitro and in planta experiments. Daily-fresh Folicur^®^ solution in SDW with final concentrations ranging from 0.005 to 0.05 µg/mL and from 0.01 to 0.2 µg/mL in the case of *F. oxysporum* and *A. solani*, respectively, were prepared prior to mixing with the WAMP-2 fragments or application on tomato leaf disks and seedlings. For both fungi, treatment at these concentrations, revealed in preliminary experiments using serial double or fivefold dilutions, resulted in a sub-fungicidal effect, not exceeding 30–45%.

### 4.4. In Vitro Assay of Inhibitory and Sensitizing Activities

The inhibitory effect of synthesized peptides on conidial germination of fungi was assessed by a microtiterplate assay [54]. Fungal conidia were added to WAMP-2-derived peptides, already dissolved in SDW, to prepare suspensions having final concentrations of 10^5^ microconidia/mL (*F. oxysporum*) or 3 × 10^4^ macroconidia/mL (*A. solani*). Resulting suspensions were incubated at 20–22 °C overnight in wells of 96-well microtiter plates. Control suspensions (with the same final concentrations of conidia) were prepared using SDW. Thereafter, germinated and non-germinated conidia (500 ones of each pathogen per treatment) were counted in treated and control suspensions using an inverted microscope. The average number of conidia germinated in these suspensions was calculated and expressed as the percent of the total number of the counted conidia. In addition, percent germination inhibition in the treated conidia, relative to germination in controls, was determined and indicated as levels of the inhibitory effect in Table 2 and Table 3. Oligopeptides were tested at final concentrations of 50, 100, 200, and 400 µg/mL, which were selected based on previous results of testing of the same WAMP-2-derived peptides for the ability to inhibit spore germination in other *Fusarium* and *Alternaria* species [44].

In vitro tests involving pathogen sensitization to Folicur^®^ EC 250 were designed under the principle of a microdilution checkerboard assay [55]. Fungal conidia were incubated in 96-well plates in mixtures of the fungicide and each of the peptides at the above-mentioned concentrations in parallel with conidia’ incubation in the solutions of Folicur^®^ or the oligopeptides, alone, used at the same final concentrations as in the mixtures.

WAMP-2 antifungal activity against *F. oxysporum* was determined earlier under the same experimental conditions [43,44].

### 4.5. Detached Seedling Wilting Test

Tomato (*Solanum lycopersicum* L.) seeds of cv. Bely Naliv, a variety susceptible to Fusarium wilt, were surface-disinfected, germinated, and sown in disinfected sand. Seedlings were grown to the stage of 4–5 true leaves as described earlier [56]. Seedlings were removed from the sand, washed with tap water followed by SDW, cut from the roots at the base of the stem, and then submerged in SDW. Detached seedlings were placed into 30-mL glass vials (three per vial) filled with a conidial suspension of *F. oxysporum* at a final concentration of 3 × 10^6^ microconidia/mL. Other portions of the detached seedlings were submerged into vials with an equivalent conidial suspension containing an aquatic solution of Folicur^®^ (0.05 µg/mL) alone, or combined with either WAMP-C or WAMP-G1 (each at a final concentration of 200 µg/mL). Control detached seedlings were placed in SDW. The seedlings were incubated in a climate chamber at 27–28°C, 150 µE^m−2^ s^−1^ (16-h photoperiod). After two-day inoculation with the pathogens, seedlings were transferred into 25% Knop solution that was refreshed daily. Wilting symptoms were observed every day for five days and photographed at 1st, 3rd, and 5th post-inoculation days (PID).

### 4.6. Tomato Leaf Disk Assay 

Leaves of 6-week-old tomato plants (cv. Volgogradsky) grown as described above (Section 4.5) were washed with tap water and disinfected by dipping in 0.35% antiformin solution for 60 s followed by thorough rinsing with SDW. Leaf disks of 2 cm in diameter were cut using a cork borer and placed into Petri plates (3 disks per plate) with 1% water agar supplemented with benzimidazole (40 µg/mL). *A. solani* macroconidia were suspended in SDW. Prior to inoculation of leaf disks, 1 mL of the suspension (5 × 10^4^ spore/mL) was mixed with 1 mL of WAMP-G1 (400 μg/mL), Folicur^®^ (0.1 μg/mL), or the fungicide combined with WAMP2-G1 (each at the indicated concentration). The resulting test mixes contained fungal spores, WAMP-G1, and Folicur^®^ at the final concentrations of 2.5 × 10^4^ conidia/mL, 200 µg/mL, and 0.05 µg/mL, respectively. A sample of the suspension in SDW with the same final concentration of conidia was used as a control. After 1-h incubation at 23–24°C, 50-μL aliquots of the treated mixes and control suspensions were pipetted onto the adaxial side of a leaf disk. The plates with inoculated leaf disks were transferred to a climate chamber and incubated as described (see Section 4.5). Early bight symptoms were visually scored at PID 9 using a six-score rating scale [32].

### 4.7. Data Analysis and Statistical Treatment

Inhibitory concentrations of the peptides providing in vitro 50% or 99% in vitro suppression of conidial germination (ED_50_, and ED_99_, respectively) were determined by the probit analysis [57] with the involvement of a linear regression. In a regression equation (*Y = ax + b*) used in probit analyses, *Y* is the probit value for 50% or 99% inhibition levels, *x* values are decimal logarithms of the correspondent concentrations, and *a* and *b* represent regression coefficients. The calculated ED_99_ values from the probit analysis were considered as the minimum fungicidal concentration (MFC), i.e., the lowest concentration of a peptide, used alone, resulting in 99.9% inhibition of conidial germination.

To reveal oligopeptide/fungicide synergy, the Limpel criterion Ee < Er [58] was determined by Formula (1):

Ee,% = (X + Y) − XY/100 < Er,% (at *p* ≤ 0.05), (1)

where Ee is the level of an expected additive effect from application of both test compounds; X and Y represent inhibition of the spore germination in % by each antifungal agent used alone; and Er is the percentage of actual inhibition obtained experimentally by co-application of X and Y.

For each of the two pathogens, microtiter plate tests with synthesized peptides included three independent experimental series. Each experiment included 5 replications of each treatment, 100 conidia per each one (*n* = 500). Experiments evaluating the sensitizing activity of the tested peptides on leaf disks and detached tomato seedlings were additionally repeated twice. Mean values, standard deviations, standard errors, as well as significant differences from controls and between treatments (*t*-test for independent variables, *p* ≤ 0.05), were calculated using STATISTICA v. 6.1 software (StatSoft Inc., Tulsa, OK, USA). Approximation of confidence values (R^2^) and regression coefficients were determined using statistical functions of Microsoft Excel 2013 (Microsoft Corp. Redmond, WA, USA).

## Figures and Tables

**Table 1:** Effective doses (ED) of the synthesized WAMP-2-derived peptides.

Peptides, mg/mL	*F. oxysporum*			*A. solani*		
	ED_50_	ED_99_ (MFC) *	R^2^	ED_50_	ED_99_ (MFC) *	R^2^
WAMP-N	0.24 ^a^	22.7	0.997	n/d	n/d	n/d
WAMP-G1	>1000	>10,000	0.936	40.0 ^c^	3980.0 ^f^	0.872
WAMP-G2	0.35 ^a^	35.1 ^b^	0.961	n/d	n/d	n/d
WAMP-C	35.2 ^b^	106.9 ^d^	0.975	0.55 ^e^	53.4 ^g^	0.998
WAMP-2	45.0 ^c^	79.0 ^d^	0.885	n/d	n/d	n/d

* ED_99_, the lowest concentration of the peptide resulting in 99.9% inhibition of conidial germination, is considered as the minimal fungicidal concentration (MFC); n/d = not determined. Significant differences (*p* ≤ 0.05) between ED values are designated by different lowercase letters (^a–g^). To calculate ED_50_ and ED_99_ of WAMP-2for *F. oxysporum*, data on antifungal activity of this peptide determined earlier under the same conditions by the same method against the same *F. oxysporum* strain [44] was used.

**Table 2:** Observed inhibitory effects on conidial germination of *F. oxysporum* by Folicur^®^ EC 250 combined with the synthesized peptides (Er) at different fractional concentrations of the components. Er values are compared to the calculated inhibition levels expected if the inhibition was additive (Ee) *.

Peptide Fractional Concentrations, µg/mL	Fractional Concentrations of Folicur^®^, µg/mL						
	0.0	0.005		0.01		0.05	
	Inhibitory Effect, %						
	Er	Er	*Ee*	Er	*Ee*	Er	*Ee*
WAMP-N							
0	*−*	6.7 ^a^*_a_*	*−*	16.9 ^a^*_b_*	*−*	26.4 ^a^*_c_*	*−*
50	12.3 ^a^	30.2 ^b^^,A^	*18.2*	45.9 ^b,A^	*27.1*	58.4 ^b^	*35.5*
100	23.1 ^b^	48.4 ^c,B^	*28.3*	58.3 ^c^	*36.1*	70.8 ^c,D^	*43.4*
200	42.7 ^c^	64.1 ^d,C^	*48.9*	69.7 ^d,C^	*52.4*	75.0 ^c,D^	*57.8*
WAMP-C							
0	*−*	6.5 ^a^*_a_*	*−*	17.8 ^a^*_b_*	*−*	25.9 ^a^*_c_*	*−*
50	4.3 ^a^	33.1 ^b,A^	*10.5*	44.2 ^b,A^	*21.0*	51.9 ^b^	*29.1*
100	6.2 ^b^	46.2 ^c,B^	*12.3*	66.1 ^c,C^	*23.8*	76.8 ^c,D^	*30.5*
200	8.6 ^c^	67.3 ^d,C^	*14.5*	83.7 ^d^	*24.9*	91.7 ^d^	*32.3*
400	11.6 ^c^	77.8 ^e^	*17.3*	87.8 ^d^	*27.3*	95.9 ^e^	*34.5*
WAMP-G1							
0	*−*	9.0 ^a^*_a_*	*−*	18.4 ^a^*_b_*	*−*	29.2 ^a^*_c_*	*−*
100	1.1 ^a^	15.2 ^b^	*10.1*	30.2 ^b^	*19.3*	45.3 ^b^	*30.0*
200	8.0 ^b^	28.4 ^c^	*16.3*	39.0 ^c^	*24.9*	49.8 ^b^	*34.8*
400	17.5 ^c^	45.4 ^d^	*24.9*	56.3 ^d^	*32.7*	64.9 ^c^	*41.7*
WAMP-G2							
0	*−*	8.1 ^a^*_a_*	*−*	19.7 ^a^*_b_*	*−*	28.0 ^a^*_c_*	*−*
50	7.0 ^a^	27.8 ^b^	*14.5*	36.4 ^b^	*24.8*	47.4 ^b^	*33.0*
100	22.4 ^b^	49.3 ^c^	*28.8*	53.1 ^c^	*37.7*	56.8 ^c^	*44.1*
200	34.1 ^c^	62.6 ^d^	*41.8*	74.8 ^d^	*47.1*	87.1 ^d^	*52.7*

* Additional results to Figure 1 and Figure 2. The difference between Er and Ee is significant at *p* ≤ 0.05 (*t*-test for independent variables) except for the one case of the additive effect (the two underlined values in one of WAMP-G1 rows), when Er exceeded Ee at *p* ˃ 0.05 (see Materials and Methods, Section 4.7). Within the column containing data for each peptide fragment, different small uppercase letters (^a–e^) indicate significant difference between Er values at *p* ≤ 0.05 (*t*-test for independent variables). The same capital superscript letters (^A–D^) within the WAMP-N and WAMP-C rows indicate statistically insignificant difference between the effects of these oligopeptides. The significant difference between Er values in the rows of the fungicide alone (0 µg/mL of peptides) is shown with subscript italic letters (*_a–c_*).

**Table 3:** Observed inhibitory effect (Er) on conidial germination of *A. solani* by Folicur^®^ EC 250 combined with the synthesized peptides, compared to the calculated inhibitory effect (Ee), when combinations had only an additive interaction*.

Peptide Fractional Concentrations, µg/mL	Fractional Concentrations of Folicur^®^, µg/mL *								
	0.0	0.01		0.05		0.1		0.2	
	Inhibitory Effect, %								
	Er	Er	*Ee*	Er	*Ee*	Er	*Ee*	Er	*Ee*
WAMP-G1									
0	*−*	6.9 ^a^*_a_*		13.1 ^a^*_b_*		28.2 ^a^*_c_*		40.9 ^a^*_d_*	*−*
100	2.0 ^a^	15.1 ^b,*A*^	*8.8 ^A^*	45.4 ^b^	*14.8*	64.7 ^b^	*29.8*	75.0 ^b^	*42.1*
200	9.8 ^b^	44.9 ^c^	*16.7*	63.1 ^c^	*21.6*	74.3 ^c^	*35.2*	78.4 ^b^	*46.7*
400	21.9 ^c^	54.2 ^c^	*27.3*	67.4 ^c^	*32.1*	78.4 ^c^	*43.9*	81.0 ^b^	*53.8*
WAMP-C									
0	*−*	6.7 ^a^*_a_*	*−*	12.9 ^a^*_b_*	*−*	28.1 ^a^*_c_*	*−*	40.5 ^a^*_d_*	*−*
100	19.0 ^a^	26.6 ^b,*B*^	*24.5 ^B^*	32.4 ^b,*C*^	*29.4 ^C^*	51.4 ^b,*D*^	*41.8 ^D^*	64.3 ^b^	*51.8*
200	31.0 ^b^	51.3 ^c^	*35.6*	54.9 ^c^	*39.9*	59.1 ^b,*E*^	*50.5 ^E^*	69.4 ^b,*F*^	*59.7 ^F^*
400	48.2 ^c^	70.8 ^d^	*51.7*	87.0 ^d^	*54.9*	92.3 ^c^	*62.8*	93.8 ^c^	*69.3*

* Additional results to Figure 3. The cases of the additive effect (Er > Ee at *p* > 0.05) are marked within the rows by the same capital italic superscript letters (*^A–F^*). Within the column containing data for each peptide fragment, different small uppercase letters (^a–d^) indicate significant difference between Er values at *p* ≤ 0.05 (*t*-test for independent variables).

**Table 4:** Amino acid sequences and molecular weights of WAMP-2 and WAMP-2-derived peptides.

Peptide Name	Amino Acid Sequence	Length, aa Residues	Molecular Weight, Da *
WAMP-N	AQRCGDQARGAKC	13 (1–13) **	1363.53
WAMP-G1	LCCGKYGFCGSG	12 (17–28) **	1194.40
WAMP-G2	CCGKYGFCGSGDAYC	15 (18–32) **	1533.73
WAMP-C	GKGSCQSQCRGCR	13 (33–45) **	1369.55
WAMP-2	AQRCGDQARGAKCPNCLCCGKYGFCGSGDAYCGKGSCQSQCRGCR	45	4658.30

* Physicochemical and structural characteristics of the oligopeptides were determined earlier [44]. ** Denotes position in the WAMP-2 molecule.

## Supplementary Materials

The following are available online at https://www.mdpi.com/2079-6382/9/12/870/s1, Figure S1: Detached tomato seedlings in (a) sterile distilled water (SDW) and SDW supplemented with WAMP-2-derived peptides: (b) WAMP-C, (c) WAMP-G1 (each at a concentration of 200 µg/mL). Seedlings were photographed after 2 days of incubation.
