# Supplementary material for: Fragments of a Wheat Hevein-Like Antimicrobial Peptide Augment the Inhibitory Effect of a Triazole Fungicide on Spore Germination of Fusarium oxysporum and Alternaria solani
Source: Antibiotics (Basel). 2020 Dec 4;9(12):870. doi: 10.3390/antibiotics9120870 (PMC7762046; doi:10.3390/antibiotics9120870)
Supplement: Supplementary file 1 [file antibiotics-09-00870-s001.pdf]

antibiotics

Article

Fragments of a Wheat Hevein-Like Antimicrobial Peptide Augment the Inhibitory Effect of a Triazole Fungicide on Spore Germination of *Fusarium oxysporum* and *Alternaria solani*

Larisa Shcherbakova [1,*], Tatyana Odintsova [2,*], Tatyana Pasechnik [1], Lenara Arslanova [1], Tatyana Smetanina [1], Maxim Kartashov [1], Marina Slezina [2] and Vitaly Dzhavakhiya [1]

[1] All-Russian Research Institute of Phytopathology, Bolshie Vyazemy, 143050 Moscow reg., Russia; beefarmer@yandex.ru (T.P.); linr-13@yandex.ru (L.A.); smetaneinatatyana@yandex.ru (T.S.); maki505@mail.ru (M.K.); dzhavakhiya@yahoo.com (V.D.)
[2] Vavilov Institute of General Genetics RAS, Gubkina Str. 3, 119333 Moscow, Russia; omey@list.ru
* Correspondence: larisavniif@yahoo.com (L.S.); odintsova2005@rambler.ru (T.O.)

Received: 14 October 2020; Accepted: 2 December 2020; Published: date

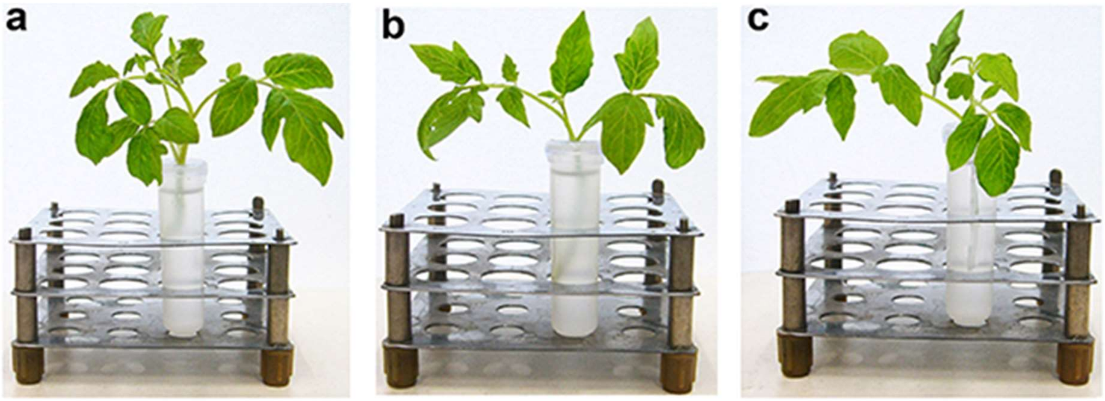

Figure S1. Detached tomato seedlings in (**a**) sterile distilled water (SDW) and SDW supplemented with WAMP-2-derived peptides: (**b**) WAMP-C, (**c**) WAMP-G1 (each at a concentration of 200 μg/mL). Seedlings were photographed after 2 days of incubation.
